# Development and Testing of a Community-Based Intervention to Address Intimate Partner Violence among Rohingya and Syrian Refugees: A Social Norms-Based Mental Health-Integrated Approach

**DOI:** 10.3390/ijerph182111674

**Published:** 2021-11-07

**Authors:** Leah Emily James, Courtney Welton-Mitchell, Saja Michael, Fajar Santoadi, Sharifah Shakirah, Hasnah Hussin, Mohammed Anwar, Lama Kilzar, Alexander James

**Affiliations:** 1 Institute of Behavioral Science, University of Colorado, Boulder, CO 80309, USA; leah.james@colorado.edu (L.E.J.); alexjms@gmail.com (A.J.); 2 Colorado School of Public Health, Environmental and Occupational Health, Anschutz Medical Campus, Aurora, CO 80045, USA; 3 ABAAD, Beirut, Lebanon; sajamichael@gmail.com (S.M.); lama.kilzar@gmail.com (L.K.); 4 Tenaganita, Petaling Jaya 46000, Selangor, Malaysia; ceciliafajar1@gmail.com (F.S.); sharifah1shakirah@gmail.com (S.S.); hasnahhussin92@gmail.com (H.H.); jfrburma@gmail.com (M.A.)

**Keywords:** refugees, social norms, mental health, intimate partner violence, intervention

## Abstract

Intimate partner violence (IPV) is the leading form of gender-based violence globally and increases during times of conflict and displacement. To reduce IPV and encourage help-seeking, a two-phase community-based intervention was co-designed with Rohingya in Malaysia and Syrians in Lebanon. Three day workshops, utilizing a social norms-based mental health-integrated approach, were implemented for women and men in each country (*n* = 148). Pre- to post-measures indicated reductions in beliefs about acceptability of violence and rigid gender norms, and improvements in mental health, functioning, coping, and self-efficacy for women and men following workshop participation. Workshop participation was also associated with increased help-seeking intent, for both mental health and IPV (victims and perpetrators). Workshops included community design of poster campaigns to address IPV, which were then tested in each setting using a randomized controlled trial in Malaysia (*n* = 240) and a matched cluster comparison in Lebanon (*n* = 260). Women in both settings found IPV less acceptable in the poster condition. Help-seeking preferences were also influenced by the poster for women and men in both countries. This participatory intervention research can provide a roadmap for use in other settings, emphasizing the value of community-generated solutions to IPV among displaced populations.

## 1. Introduction

### 1.1. Global Prevalence of Intimate Partner Violence

Intimate partner violence (IPV) is the leading form of gender-based violence globally [1]. IPV includes physical, sexual or psychological harm by a partner, and occurs in every community, regardless of socio-economic status, race, ethnicity, or religion [2,3]. The most comprehensive study of IPV to date, the World Health Organization (WHO) Multi-Country Study on Women’s Health and Domestic Violence, surveyed almost 25,000 women in 10 low- and middle-income countries (LMICs) in the early 2000s. Of ever-partnered women, between 15 and 71% reported lifetime experience of IPV in the form of physical and/or sexual violence, with most sites reporting between 30 and 60% (or 15 and 30% within the last 12 months). Results indicate that much of the violence women experience from intimate partners is frequent and severe [1,4,5].

### 1.2. Increased Risk of Intimate Partner Violence during Conflict and Displacement

Rates of IPV are often higher for those experiencing conflict, including displaced populations [6,7]. For example, Syrian and Lebanese women engaged in psychosocial programming reported 77% lifetime prevalence of IPA, 52% in the last 12 months [8]. In pilot research with Rohingya in Malaysia and Syrians in Lebanon (an initial phase of the current study), rates of IPV were similarly high. Just over 50% of Rohingya women in Malaysia, and almost 30% of Syrian women in Lebanon, reported being ‘punched, kicked or beat up’ in the last 12 months [9,10].

Risk factors for IPV include factors commonly associated with conflict and displacement. Research in Colombia, Côte d’Ivoire, and Lebanon suggests that environmental stressors, including economic strain, may overwhelm coping ability and contribute to IPV [8,11,12]. Rohingya in Malaysia and Syrians in Lebanon perceived the following chronic stressors as increasing risk of IPV: fear of arrest, insecure legal status, lack of private shelter, lack of employment opportunities, limited access to health care and educational opportunities [9,10].

### 1.3. Barriers to Help-Seeking

IPV is believed to be widely underreported, with many survivors and perpetrators of IPV not reaching out for help or accessing services [13,14,15,16]. For example, among married women experiencing IPV in Myanmar, 93% did not seek help [17]. In a study of help-seeking among victims of IPV in 31 LMICs, just under 35% engaged in help-seeking, with the majority of these (62%) reaching out to family, and less than 5% engaging with formal institutions [18]. Such findings are consistent with exploratory data collected by this team, including low rates of help-seeking among Rohingya and Syrians, and a preference for informal help-seeking channels such as family members [9,10].

Low rates of help-seeking are likely a result of specific barriers, including fear of accessing services due to insecure legal status, shame, expectations of victim blame, and social norms discouraging help-seeking, especially outside of the immediate family [19,20,21]. Additionally, displaced populations often have few legal protections in their host country and may experience language and other barriers unique to their situation [22,23,24].

### 1.4. Intimate Partner Violence, Social Norms and Gender Roles

Rigid gender roles and other social norms emphasizing what is considered acceptable behavior for men and women can increase risk of IPV, exacerbate victim blame, and undermine help-seeking [18,25,26,27,28,29,30,31]. Research indicates that certain norms, such as beliefs that a man has the right or responsibility to ‘discipline’ his partner, that a man’s abusive behavior is indicative of love, and that it is a woman’s duty to tolerate abuse, have been associated with increased risk of IPV and decreased likelihood of help-seeking [25,32,33,34]. Rigid gender roles seem to underpin many of these social norms, including patriarchal expectations that men should be controlling and women submissive [10,11,35,36,37]. Increasingly, perspectives from practitioners and researchers indicate that IPV interventions should focus on addressing social norms contributing to acceptability of IPV, including rigid gender roles and stigma around help-seeking [8,25,27,38,39,40,41]. Researchers have also emphasized that additional work is needed to address gaps in understanding links between gender attitudes, partner violence and mental health, and to test related interventions [27].

### 1.5. Mental Health and Intimate Partner Violence

Mental health is associated with IPV and help-seeking in several ways. First, IPV victimization often results in poor mental health [42]. In a recent systematic review among girls and women in LMICs, IPV and other forms of gender-based violence were consistently associated with adverse mental health outcomes, including depression and post-traumatic stress disorder (PTSD) [43]. Additionally, mental health symptoms, such as depression, may undermine help-seeking among victims of IPV [22,44]. Furthermore, depression, PTSD, substance abuse, and other mental health challenges appear to increase the risk of perpetrating IPV [45,46,47,48]. Such research indicates that intervention approaches to IPV should focus on addressing the linkages between mental health and IPV, including how symptoms might influence help-seeking and/or abusive behavior. One approach is to integrate mental health and psychosocial support (MHPSS) content into IPV interventions, including community-based psychoeducational workshops. A mental health-integrated approach to intervention has shown promise in other settings, including with displaced populations in Haiti and Nepal during disaster preparedness workshops [49,50]. Furthermore, IPV interventions focused on enhancing help-seeking, including informal social support, have shown promise in terms of mental health impacts [51]. Some research suggests that peer-support approaches with mental health components may also be useful in working with male perpetrators of IPV, including those with a history of trauma exposure [52].

### 1.6. Interventions for Intimate Partner Violence: Group-Based Models

In a review of IPV interventions [53], group-based models, including workshops, have shown promise in addressing IPV, especially those focusing on social norms and acceptability of violence. Furthermore, the review indicated that many successful IPV interventions in LMICs include group workshops aimed at challenging gender role norms and building new skills for coping, communication, and conflict resolution. This includes group programming involving both women and men in violence prevention, successfully resulting in attitude change, in addition to social support and mental health impacts [51,54].

### 1.7. Interventions for Intimate Partner Violence: Messaging Campaigns

Poster-based messaging campaigns are widely used for health-related purposes across contexts, especially in LMICs. This includes use of IPV posters, which often aim to encourage attitude shift and/or a behavioral response (e.g., call a hotline) [55,56,57]. IPV poster campaigns are increasingly common in LMICs, associated with global movements such as ‘16 Days of Activism Against Gender-based Violence’ and ‘Orange the World to End Violence Against Women’. With so many resources put into campaigns that utilize posters, it is important to understand what type of impact is possible with this type of intervention. While research suggests that IPV posters can be efficacious, influencing attitudes and motivating help-seeking [58], rigorous experimental research on poster campaigns, including in LMICs, is limited [57]. Researchers have cited a need to examine health communication campaigns in diverse cultural settings, including LMICs [57,59]. 

### 1.8. Participatory Interventions Addressing Social Norms and Mental Health

Community participatory methods are a key element of ethical research with refugee communities [60] and are critical for the development of effective interventions that encourage reciprocal knowledge transfer [61]. Co-creation of research methodology and intervention curricula, including through use of community advisory boards [62], can empower community members and result in more effective, sustainable, participant-centered approaches better suited to the cultural context. Participatory approaches can also build trust between researchers and community members, increasing participant engagement and enhancing effectiveness of interventions [61]. Participatory models can align with social norms role-modeling approaches, such that affected community members serving as intervention facilitators and researchers can serve as prosocial role models for their peers. Participatory methods are especially well suited for humanitarian settings with displaced populations that are often disenfranchised, providing an opportunity to promote equity and address power imbalances [63].

Community-based participatory research methods have been used effectively to develop culturally-adapted IPV tools and interventions [64,65], including for people with mental health difficulties [66]. A review mental health and psychosocial interventions utilizing participatory approaches with displaced populations [67] revealed benefits of this approach. Consistent with others, authors of the review emphasized a need for increased research to create evidence for community-based work, including of the type described in this manuscript [55,56,68,69].

### 1.9. Current Study

More rigorous multi-site research is needed to contribute to understanding what types of IPV interventions are effective in specific LMIC contents, including participatory workshops, messaging campaigns, and interventions involving men [55,56]. The purpose of the current study, targeting women and men Syrian refugees in Lebanon and Rohingya in Malaysia, is to create and test two community-based IPV interventions in two unique settings. This includes examining the impact of (1) participation in a 3 day interactive IPV workshop (*n* = 148) and (2) exposure to community designed IPV poster campaigns (*n* = 500) that conform to recent best-practice guidance for behavioral science-informed health campaigns [69].

Data reported here were collected between 2017 and 2018, an especially tumultuous time for Rohingya and Syrians globally. During this period, violence against Rohingya increased in Myanmar, resulting in large population displacement, while living conditions in host nations, including Bangladesh, Malaysia and elsewhere, continued to deteriorate [70,71]. Throughout the period 2017–2018, the conflict in Syria continued to its 7th year, with Syrians representing the largest forcibly displaced population worldwide by the end of 2018 [72]. Syrians, like Rohingya, found host countries increasingly inhospitable, with many, including Lebanon, enforcing increasingly restrictive protocols limiting access to basic services and interfering with daily living [73].

The research highlighted in this manuscript was part of a larger study, including an initial exploratory phase consisting of interviews and focus group discussions (FGDs). Results from this exploratory phase were used to inform workshop curricula development [9,10]. This paper focuses on workshop implementation and assessment (Study 1) and poster campaign development and testing (Study 2). Methods and results for each study are detailed separately in the following section. Each phase of this research was approved by three separate ethical review boards—in the U.S., Malaysia and Lebanon. Please see details in the ‘Institutional Review Board Statement’.

## 2. Study 1. Workshop Implementation and Assessment

### 2.1. Study 1. Materials and Methods 

#### 2.1.1. Workshop Curricula Development and Faciltiator Training

Workshop curricula manuals were developed collaboratively by cross-national teams, including the Principal Investigators (PIs) and partner organization teams (ABAAD and Tenaganita). Manual content was informed by a literature review incorporating GBV and mental health materials used in refugee contexts, including intervention materials from ABAAD [38,74,75], Tenaganita, and prior intervention work by the PIs [49,50]. Content was also informed by interviews and FGDs conducted in each setting during a prior exploratory phase, designed to identify community-specific norms related to IPV in each setting [9,10].

Manuals were written for use by both men and women participants, with some sections designated to be used specifically with men or women. The Lebanon manual was translated into Arabic. The Malaysia manual was orally translated into Rohingya language via audio-recording, to be accompanied by a written glossary of Rohingya terminology (to accommodate lack of formal written Rohingya language).

Following the development of initial drafts of manuals, Community Advisory Committees (CACs) were formed, including Syrian/Rohingya community leaders, such as mokhtars, religious leaders, civil society leaders, and teachers. CAC Manual Development Workshops were held over 1–2 days in each setting to solicit feedback to ensure the best fit for local realities. Manuals were also used in methods development and training workshop with facilitators. Final manuals, revised based on this feedback, are publicly available in English, Arabic and Burmese, by request to authors and at MHPSS.net [76,77].

Two men and two women facilitators were recruited from local Syrian and Rohingya communities. In each location, 5 day methods development/training sessions including role-playing were held to elicit team input about the curriculum and interview tools, and to prepare team members for implementation.

#### 2.1.2. Workshop Participants: Sampling, and Recruitment

In both settings, women and men refugee community members aged between 18 and 60 were eligible to participate in workshops. Participants were not screened for personal experience with IPV; however, based on prevalence rates in other research, including data collected during an earlier phase of this research, it was assumed that many participants had such experiences. Service providers/community leaders, aged 18 and older, with experience working with the specific communities in each location, were also eligible to participate in separate workshops.

Malaysia. Seventy-four Rohingya community members residing in Malaysia participated in the workshop phase of the project during the period August–November 2017. This included two women’s groups (29 participants), two men’s groups (30 participants), and one service provider/community leader group (15 participants). Participants in Malaysia were primarily sampled from two of 14 identified communities in Gombak district. Participants were recruited through door-to-door visits by Rohingya members of the research team using a recruitment script, with one adult (a man or women) invited from each household.

Lebanon. Seventy-four participants were involved in a parallel workshop phase conducted in Lebanon in the period September–November 2017. This included two women’s groups (30 participants), two men’s groups (30 participants), and a service provider/community leader group composed of a mix of Lebanese, Syrian, and Palestinian men and women (14 participants). Syrian community member participants in Lebanon were primarily sampled from the El Marj community in Bekaa Valley. Potential participants were recruited by phone or in person at partner organization activities by Syrian and Lebanese members of the team, using a recruitment script.

In both settings, for safety reasons, only one member of a couple or household was invited to participate. Those who expressed interest participated in a verbal informed consent process in a private area.

#### 2.1.3. Workshop Implementation

In line with social norms role-modeling approaches, 3 day community workshops were facilitated by Rohingya and Syrian refugee community members who were gender matched and from similar backgrounds to those of participants. The aim of this approach was to increase participant receptivity to content presented by in-group members. Workshops were held in easily accessible and non-stigmatizing public settings (the Marj public library in Lebanon and a local Rohingya organization office in Malaysia). Childcare was provided on site at the workshop locations. During the workshops, participants were given meals and workshop materials (e.g., supplies and manual). In both settings, a stipend was provided to compensate for transportation and potential missed work.

Day 1 of the workshop opened with a pre-workshop knowledge survey and group cohesion-building activities. This was followed by psychoeducation about mental health related to conflict and displacement and discussion about gender roles. Starting on the first day and continuing throughout the workshop, results of exploratory data collection in each community were incorporated to set a foundation regarding the importance of IPV interventions, and to spark discussion.

Day 2 included discussion about gender norms and IPV, including related role-plays and other activities. Participants also practiced coping skills to reduce stress, discussed legal protections and religious perspectives on IPV, and impediments to help-seeking. Finally, the concept of social norm messaging was introduced and the group viewed and critiqued a series of anti-IPV campaigns developed by other actors.

Day 3 was devoted to work in small groups to develop community-based campaigns designed to discourage perpetration of IPV and encourage help-seeking. Although various campaign types were discussed, group participants developed poster campaigns, both because these are widely used intervention tools [57,58,59], and because evaluating posters was feasible within the scope of this project. Groups developed posters with visual and written messaging and presented their campaign to peers. This activity aimed both to develop campaigns for use in the subsequent poster testing phase, and to empower participants to use their expertise about IPV and social norms messaging to benefit their peers.

#### 2.1.4. Interviews

Participants were interviewed by gender-matched research team members (trained Syrian and Rohingya community members) immediately before and two weeks after participation in the workshops. Interviews were completed in private areas in participant households or community centers using Qualtrics survey software on tablets. The research team provided on-site support as needed, and referrals for all to GBV and mental health services. Participant compensation during interviews was in line with norms set by partner organizations and others doing similar work in each context. Interview measures used in Lebanon were translated to Arabic and back-translated by a separate translator to ensure accuracy. In Malaysia, due to the lack of standardized written Rohingya language, audio recordings were used to translate materials from English to Rohingya and then to back-translate to English.

Measures are described in Table 1. Measures were selected based on having been used across a wide variety of cultural contexts and settings, including with populations similar to those described here. Measures were pilot tested and revised in Lebanon and Malaysia during the initial exploratory phase of this project. Cronbach’s alpha is provided for standardized measures.

#### 2.1.5. Analysis Approach

Because Shapiro–Wilk tests indicated data were not normally distributed, change from pre- to post-workshop participation was analyzed using the non-parametric Wilcoxon Matched-Pairs Signed Rank test. For brevity’s sake, multiple-item measures are presented as composite scales when possible. Participants with missing outcome data at either time point were removed from those specific analyses. In Malaysia, for most outcomes no more than 1–2 cases (2%) were missing, with a few with up to 7% missing. In Lebanon, missing data ranged from 0 to 5% for the majority of key variables, with a few at 8–10%. Items that may have created discomfort, leading to higher rates of missing data in Lebanon (gender relations scale items: *My community thinks/I think that a woman cannot refuse to have sex with her husband*) had 27%/23% missing data at pre-intervention interviews. However, following additional interviewer training, data were no longer missing at higher rates in post-intervention interviews. Gender relations scale analyses run with mean imputation did not significantly change results. With the sample sizes used, power to detect a within-subject difference with a Cohen’s effect size of 0.2, 0.35, and 0.5 was 38%, 82%, and 98%, respectively (for both countries).

### 2.2. Results

#### 2.2.1. Demographics and Prevalence of IPV 

Descriptive statistics for the workshop sample are presented in Table 2.

#### 2.2.2. Outcomes

Participants in the workshop demonstrated significant change in outcome variables from pre- to post-intervention (see Table 3). In both countries, participants demonstrated reduced perceived acceptability of violence, and endorsed more equitable gender norms at the individual level (*I believe that…*) following participation in the workshop. In Malaysia, participants also reported more equitable gender norms at the community level (*My community believes that…*). However, in Lebanon, participation in the workshop was associated with reduced perception of community-held equitable beliefs.

For women and men in both countries, intent to seek help increased significantly following the workshop, including for mental health-related help-seeking, and for help-seeking if being victimized or perpetrating IPV.

In both countries, participants demonstrated reduced emotional distress and functional impairment, and increased use of adaptive coping skills following workshop participation. They reported reduced use of arguing/yelling and beating children as coping mechanisms to reduce stress and tension, and in Lebanon, also reported reduced use of physical violence as a coping mechanism. Participants in both countries reported increased self-efficacy, and in Malaysia, increased community efficacy and social cohesion.

Gender-wise analyses were conducted for all workshop-related outcome variables. For Malaysia, all results for both and women were the same as the combined results except for the community scale (perception of what community believes) and social cohesion which were not significant for men. In addition, the coping item ‘*I’ve been hitting my children to release tension*’ was significant for women but not men. All tests run separately for community member participants without service provider participants and were statistically significant except for social cohesion.

For Lebanon, by gender results were the same as combined results, except for community ideas about gender relations which was not significant for women and was trend-level for men (*p* = 0.075). All results for community member participants without service provider participants were the same as combined results.

#### 2.2.3. Participant Satisfaction, Perceived Impact, and Reactions to Research Participation

Immediately after the workshop, participants in both countries completed satisfaction surveys. In Malaysia, more than 95%, and in Lebanon, more than 90%, of participants reported that they agreed or strongly agreed with all items assessing satisfaction with the workshop.

During the post-intervention interviews conducted two weeks after the workshops, most participants reported that they agreed or strongly agreed that the workshop had helped them to *cope with distress* (Malaysia: 95.8%; Lebanon: 85.2%), to *feel that I am contributing to improve the status of my community* (Malaysia: 100%; Lebanon: 98.7%), and that *after developing the messaging campaign I feel that I am an active member of my community* (Malaysia: 100%; Lebanon: 98.6%). In Malaysia, 94% reported that they had *shared content and skills learned in the workshop with others*, whereas in Lebanon, more than half (58.2%) indicated this was the case.

On the Reactions to Research Participation Questionnaire (RRPQ), almost all participants reported that they agreed or strongly agreed with the statements, *Knowing what I know now, I would participate again* (100% in Malaysia; 95.9% in Lebanon) and *I found participating to be beneficial to me* (100% in Malaysia; 97.3% in Lebanon), and almost all disagreed or strongly disagreed that *Participating was inconvenient for me* (98.6% in Malaysia; 94.6% in Lebanon).

## 3. Study 2. Poster Campaign Development and Testing

### 3.1. Study 2. Materials and Methods

#### 3.1.1. Poster Development

**Figure 1:**
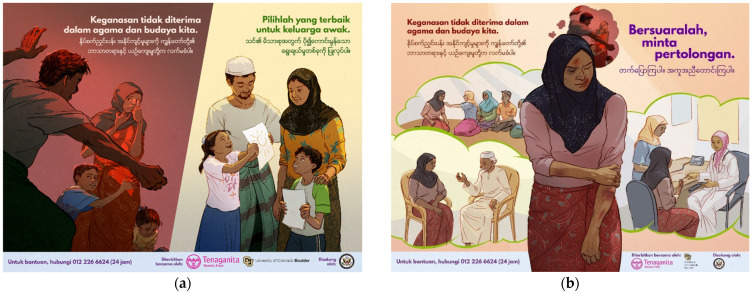
Malaysia campaign posters, final versions after community feedback. (**a**) English translation, Addressing IPV Poster: Main Slogan: *Abuse is Not Acceptable in our Religion or Culture*. Sub-slogan: *Make a Better Choice for Your Family.* (**b**) English translation, Help-seeking Poster: Main Slogan: *Abuse is Not Acceptable in our Religion or Culture*. Sub-slogan: *Break the Silence and Ask for Help*. Artist: Charis Loke, used with permission.

**Figure 2:**
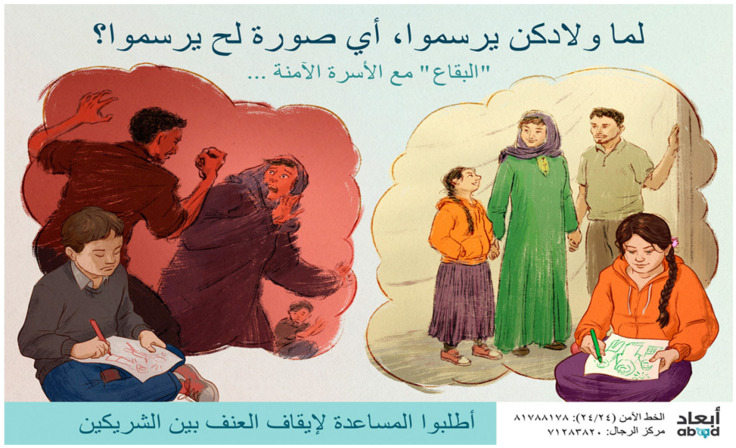
Lebanon campaign poster, final version. English translation, Main Slogan: When Your Children Draw Their Family, Which Picture Will They Draw? Sub-slogan: Al Bekaa Supports Safe Families. Ask for Help to Stop Intimate Partner Abuse. Artist: Charis Loke, used with permission.

In each setting, posters designed by community members during the workshops were reviewed by multi-national project teams, including local community members. Poster designs that represented themes that were the most common in each setting and had a clear message were selected for final work-up by a professional artist. In Malaysia, the team elected to develop two posters, one focused on discouraging family violence, designed to be viewed by men, and another encouraging various sources of help-seeking, designed to be viewed by women. ln Lebanon, the team developed a single poster designed both to discourage violence and encourage help-seeking, to be viewed by both women and men. Interestingly, in both countries, posters focused on families and encouraged prosocial norms (see Figure 1 and Figure 2).

Poster images and slogans for both settings were rendered by an experienced Malaysian artist and were pilot tested with community members. Based on community feedback, poster components were modified such as clothing type (length and color), color of head coverings, type of facial hair, positioning and facial expression of figures. In Malaysia, 84% and in Lebanon, 94% of respondents in the final sample indicated that the people in the poster campaign could be “members of your own community” *somewhat, very much or extremely*.

#### 3.1.2. Poster Testing: Research Design

In Malaysia, 240 Rohingya community members residing in Gombak District (121 women and 119 men) participated in a randomized controlled trial design to test the impact of two community-generated IPV posters. Women and men were randomly assigned to poster or no poster conditions using random number generation through Excel, conducted by the PIs. In the poster condition, women viewed the poster encouraging help-seeking and men saw the poster discouraging family violence.

In Lebanon, 260 Syrian community members residing in Bekaa Valley (131 women and 129 men) participated in a cluster comparison to test the impact of one community-generated IPV poster. Rather than using individual-level randomization, communities were randomized to condition, reducing the likelihood of contamination resulting from neighbors talking to each other about the campaign. Potential for contamination was identified as a significant liability because refugee participants live in close proximity and are prone to communicate frequently about NGO activity (even when asked not to share) due to worry that aid may be distributed unfairly. To prevent such contamination, six hundred households in areas close to the El Marj community were identified across nine community clusters for potential sampling. A sampling survey with key informants (‘Shawish’ community leaders) from each cluster was conducted to select six clusters that were well matched on key demographic variables and endorsement of IPV-related norms. Clusters were then randomized (using a random number generator in Excel, conducted by the PIs) into poster and control (no poster) conditions. In the poster condition, women and men viewed the same help-seeking/family violence poster.

A total of 265 participants were interviewed, with five removed from analyses because their responses to questions regarding the basic content of the posters indicated they did not understand the campaign. Two participants missing condition tags were removed from outcome analyses.

#### 3.1.3. Participants: Sampling and Recruitment

Participants in both Malaysia and Lebanon were sampled from the same overall sampling frames (Gomback district in Malaysia and El Marj in Lebanon) used in Study 1, but different areas within the frame were targeted for each study. For the Study 2 poster phase of the project, all households within the sample frame were invited to participate, although some eligible participants were not at home at the time when the study team visited.

At each Rohingya or Syrian dwelling in the sampling frame, either an adult woman or man (alternating by household, with interviewer and participant gender matched) was invited to participate in the project by a member of the research team. Dwellings were given numbers and descriptions (and in Malaysia geotagged) during the initial visit. All participants during the initial visit were consented. For those in the poster conditions, the poster was shared for two full minutes in the presence of the interviewer. This exposure duration is consistent with what is used in experimental paradigms and is realistic for viewing an IPV poster in a naturalistic setting [58,86,87]. Given that posters were intended to be viewed independently, and for visuals to function separately from the text for those with low literacy, the interviewer did not read the poster text to the participant. While viewing continued, participants were asked questions about the poster to assess understanding of the message and perceived impact. Those who were in the no-poster control condition were only asked to engage with the consent document. In both conditions, participants were told that another interviewer would be visiting them in a few days to ask different questions (implying an unrelated study).

Several days later, a separate interviewer (for purposes of blinding to poster condition) came to the home and spoke to the same participant, this time to conduct the interview containing the outcome measures. At the end of the interview, participants were asked if someone had previously visited their home to show them a poster, which served as a validation check to ensure that participants in the poster condition had viewed a poster and those in the control condition had not.

All participants were provided with contact information for partner organizations (Tenaganita and ABAAD) and other service providers.

#### 3.1.4. Interviews 

Study 2 interview measures were translated to local language using the same procedure used in Study 1. See Table 4 for Study 2 measures. 

#### 3.1.5. Analysis Approach

Scales for both countries were calculated as means of scale items and primarily analyzed by linear regression (except for prevalence of IPV and acceptability of IPV, where binary items were summed and analyzed by logistic regression). Individual items were analyzed by logistic regression for binary outcomes and by cumulative logit model for ordinal items. To assess differences between control and experimental groups on demographic variables, Fisher’s exact test was used for nominal variables and *t*-test for continuous variables.

In Malaysia, the impact of posters was analyzed using separate regression equations for men and women (because they saw different poster campaigns). For Lebanon (cluster comparison, men and women saw the same poster campaign), the impact of the poster was analyzed with men and women together in a single regression equation. Each dependent variable was first probed for a poster condition x gender interaction; if it was not statistically significant, condition and gender main effects were assessed. Because intraclass correlation levels were low (<0.1) and cluster random intercepts did not improve model fits, sample clustering was not explicitly modeled.

With the sample sizes used, power to detect a between-group effect of the poster with a Cohen’s effect size of 0.2, 0.35, and 0.5 was 19%, 48%, and 78%, respectively, for Malaysia, and 36%, 80%, and 98%, respectively, for Lebanon. It is important to note that while intra-class correlations were not especially large, it is nevertheless possible that power realized was lower than these figures for the Lebanon study because intraclass correlations were still not zero and because there were a relatively low number of clusters.

For all outcome measures, data were missing for less than 5% of participants, except for the help-seeking scenario question (*Do you think the woman will want to seek help?*), which was missing for 10.9% of men in Malaysia. Missing data were excluded, except for the individual beliefs about gender relations scale, where missing values were replaced by subject means (no more than 2.7% of the data for any item in the scale was missing).

### 3.2. Results: Study 2

#### 3.2.1. Demographics, Prevalence of IPV and Help-Seeking

Sample demographics are presented in Table 5. For both countries, intervention and control group participants were compared to assess the extent to which randomization (individually in Malaysia and by community in Lebanon) was successful and groups were well matched. In both Malaysia and Lebanon, there were no significant differences between poster and control groups on any demographic variables. There were also no significant differences in mental health symptoms.

In both countries, the poster group reported similar prevalence of IPV to the control comparison group, except for being pushed, shoved, or slapped by their partner which was reported by a larger percentage of Syrian participants in the poster condition than in the control group (19.3% vs. 9.8%, *p* = 0.04). This effect was driven by women (only one man reported that his partner did this to him).

In Malaysia, because the percentage of men partnered/currently living with a partner was notably smaller than the percentage of women currently living with a partner, the study team ran subgroup analyses. As expected, IPV prevalence among partnered men was higher than in the full sample of men (reported in Table 5). However, examining outcomes for the subgroup of currently partnered men did not change any findings that were significant for the full sample of men. As a result, results for the full sample are reported, including in Table 5 and Table 6.

#### 3.2.2. Outcomes

Results are presented in Table 6. In Malaysia, Rohingya women who were randomly assigned to view the help-seeking poster reported significantly reduced acceptability of violence in comparison to those who did not view the poster. In Lebanon, there was a significant poster condition by gender interaction, such that women who reviewed the poster reported reduced acceptability of violence in comparison to those who did not view the poster (OR = 0.19, *p* = 0.035, 95% CI: 0.04–0.89) while men who viewed the poster reported higher acceptability of violence compared to those who did not (OR = 2.12, *p* = 0.04, 95% CI: 1.05–4.29).

In Malaysia, there was a trend effect such that women were more likely to endorse more egalitarian views of gender relations and roles.

Women in Malaysia reported significantly higher relationship self-efficacy (ability to address conflict in relationships) after viewing the poster compared to those who did not. In Lebanon, there was no condition by gender interaction; however, in an analysis controlling for gender, there was a significant effect of the condition such that those who viewed the poster reported higher relationship self-efficacy than those who did not.

In Malaysia, there was a trend result for women in the poster condition such that more women indicated that they would seek help if being abused compared to those who did not see the poster.

Intent to access particular sources of help did vary by poster condition in both settings. In Malaysia, women who viewed the poster were more likely to report that they would seek help from religious leaders and social institutions. In Lebanon, there was a significant condition by gender interaction for help-seeking from family members, such that women were more likely to seek help from family members in the poster condition and men less likely (though gender-wise analyses did not reach significance). On the other hand, men in the poster condition were significantly more likely to seek help from partners’ family members (OR = 2.59, *p* = 0.009, 95% CI: 1.28–5.24), while women were less likely (OR = 0.44, *p* = 0.04, 95% CI: 0.20–0.96).

There were significant results in Lebanon, for the specific investigator-created additional item added to the gender relations measure, *those experiencing IPV should keep it to themselves.* Controlling for gender, participants were less likely to agree with this in the poster campaign condition.

There was a trend for women in Malaysia by condition in terms of whether they thought the woman described in the IPV scenario would want to seek help, with those in the poster condition more likely to say yes.

In Malaysia, women were less likely to say that they *wouldn’t get involved* if a couple were experiencing IPV in the poster condition. In Lebanon, when controlling for gender, there was a trend such that those in the poster condition were less likely to say that they would not get involved.

In Lebanon, there was a significant condition by gender interaction such that men in the poster condition were more likely to say they would speak to the wife in relation to an IPV incident, compared to those in the control condition (OR = 4.10, *p* = 0.0003, 95% CI: 1.91–8.80). Additionally, when controlling for gender, there was a significant effect of condition such that those participants in the campaign condition were more likely to speak to the husband in the scenario.

On the Reactions to Research Participation Questionnaire (RRPQ), almost all participants reported that they agreed or strongly agreed with the statements, *Knowing what I know now, I would participate again* (94.2% in Malaysia; 92.6% in Lebanon) and *I found participating to be beneficial to me* (91.3% in Malaysia; 90.4% in Lebanon), and almost all disagreed or strongly disagreed that *Participating was inconvenient for me* (93.8% in Malaysia; 94.6% in Lebanon).

## 4. Discussion

### 4.1. Studies 1 and 2

In two linked studies, the research team assessed (1) an innovative in-person workshop model integrating mental health and social norms, and (2) poster campaigns developed by participants in the workshops. Parallel studies were conducted in two settings (Malaysia and Lebanon) with two displaced populations (Rohingya and Syrians). Together, results of these two studies provide encouraging initial evidence for the benefits of community-engaged workshop and poster campaign interventions in reducing the acceptability of intimate partner violence and promoting help-seeking (among other outcomes).

Both interventions were effective in various ways, and across contexts, while also revealing gender and contextual differences that raise considerations for future research. Results suggest that these intervention approaches are relevant both for women (most commonly victims of IPV) and men (most commonly perpetrators) and therefore address a documented need for intervention approaches utilizing social norms, integrating mental health, and targeting both victims and perpetrators [27,89,90,91]. 

### 4.2. IPV Prevalence and Willingness to Report

Not surprisingly perhaps, reported rates of IPV were high in both settings, and similar to rates reported elsewhere, including in research with other displaced and conflict-affected populations [8].

In Study 2, while poster and control group participants in both countries were not significantly different on demographic and mental health variables, suggesting that randomization and cluster matching was generally successful, there were differences in reported rates of IPV in Lebanon on one item. More than twice as many participants in Lebanon endorsed being pushed, shoved, or slapped by their partner in the poster condition compared to the control condition (an effect driven by women). It is possible that this finding reflects a baseline difference between poster and control samples, such that prevalence of IPV was higher for those in the poster condition from the outset. However, another possibility is that viewing the poster increased participant willingness to report IPV, a result that may be likely in light of evidence that IPV is notoriously underreported [92]. If so, the poster may have helped women participants in Lebanon to feel more comfortable reporting true levels of IPV (whereas those in the control condition may have underreported). More research is needed to explore these possibilities.

### 4.3. Acceptability of IPV, Gender Roles/Norms

Both workshops and posters were designed to impact beliefs in the acceptability of IPV, with the underlying assumption (substantiated by other research [25,32,33,34]), that a change in beliefs about the acceptability of IPV would reduce actual IPV risk. Given this, much of the workshop content focused on challenging beliefs and rigid gender norms. In both settings, and for both genders, workshop participants showed significant pre- to post-intervention benefits, including a decrease in acceptance of IPV and an increase in personal beliefs associated with more egalitarian gender norms, and less rigid gender roles.

In Malaysia, workshop participation was also associated with increased perception that the (Rohingya) community endorses more gender-equitable norms. However, the opposite effect was found in Lebanon, such that participants perceived community norms as less equitable following workshop participation. Although the intention of the workshop was to help to shift both individual and perception of community norms toward increased equitability, it is also possible that the workshop, which entailed reflection on data collected in their community showing inequitable beliefs and high rates of IPV, awakened Syrian participants to the reality of such norms in their community. This may not be detrimental, especially since individual level attitudes showed predicted change, and in fact, increased awareness of problematic community norms may represent a necessary stage in moving toward positive change. Overall, the workshop results indicate that this type of social norms-based mental health-integrated workshop can influence personal beliefs and perception of community beliefs.

Additionally, results from posters, similarly designed to address attitudes about IPV using social norms-based approaches, indicate that women who viewed the posters found IPV less acceptable in both settings. Furthermore, in Malaysia, there was a trend effect for women in the poster condition towards endorsement of personal belief in more equitable gender norms. Of note, in Lebanon, men showed an unexpected reverse effect in which those who viewed the poster reported that violence was more acceptable compared with those who did not. More research is needed to understand whether this represents a ‘backlash’ effect, such that viewing the poster created an opposite reaction in men, or whether effects can be explained by methodological issues (potential baseline differences between matched clusters on unknown variables). If the former, it is possible that men may have felt threatened or blamed by campaigns (in which men are depicted as the perpetrator) and became defensive as a result. If so, this represents an important warning regarding poster campaigns for IPV, which in some populations, may create unexpected and undesirable effects. These results are in line with a study that found a decrease in men’s attitudes about the severity of IPV after they engaged with a prevention campaign [93]. Authors of that study suggested that resentment and victim blame may have contributed to men rejecting campaign messages.

### 4.4. Help-Seeking and Help-Giving

Consistent with other research [18], reported rates of help-seeking in both settings indicate that many people do not seek help for IPV (whether the victim or perpetrator). For women and men workshop participants in both countries, intent to seek help increased significantly following the workshop, including for mental health-related help-seeking, and for help-seeking if being victimized or perpetrating IPV. 

In Malaysia, women who viewed the poster were (at trend level) more likely to report that they would seek help for IPV and were more likely to state that a hypothetical woman described in an IPV vignette would want to seek help. The poster also influenced the type of help-seeking response. Women who viewed the poster in Malaysia were more likely to seek help from religious leaders and social. In Lebanon, women in the poster condition were more likely to seek help from family members, and men were more likely to seek help from their partner’s family. However, both were less likely in the poster condition to seek help from the man’s family members, a puzzling result that should be explored further. Participants who saw the poster were also less likely to agree that people experiencing IPV should keep it to themselves.

Furthermore, in both settings, posters affected reported intention to help others experiencing IPV. Participants in Lebanon and Malaysia (women only) were less likely to indicate that they would *not* get involved in a community incident of IPV. In Lebanon, both women and men who viewed the poster were more likely to indicate that they would speak with the husband (perpetrator) in the scenario, and men were more likely to talk with the wife. Men’s intention in speaking to the wife is not clear; although the intention could be to provide support, these findings could also imply that men perceive the women is partly to blame.

These results suggest that poster content should be chosen deliberately and directly linked to the specific source of help-seeking that is being encouraged. In Malaysia, the poster that women saw featured several potential help-seeking sources, including family, friends, religious leaders and services providers. Posters that men saw in Malaysia, and that women and men saw in Lebanon, were not explicit in terms of specific sources of help-seeking. However, results suggest that even more general IPV poster campaigns may encourage help-seeking, including within the extended family. Practitioners should consider how to capitalize on informal help-seeking within existing social networks, including potentially offering ‘bystander training’ to those likely to be perceived as informal sources of help such as family and community leaders.

### 4.5. Problem-Solving Efficacy and Social Cohesion 

In both settings, self-efficacy improved for both genders following the workshop, indicating that participants felt more confident that they could take steps and find solutions for relationship conflict. Much of the workshop content focused on solution-oriented approaches to IPV, including community-based initiatives, so this may explain this change.

In Malaysia, all workshop participants also showed benefits in regard to collective efficacy (*my community can find solutions*…), and women reported a significant increase in social cohesion. This suggests that the group intervention helped participants in Malaysia to consider how to collectively address IPV-related problems. Interestingly, there were no significant improvements in collective efficacy or social cohesion in Lebanon. Additional research is needed to further investigate these null results, including the possibility that context specific factors may have eroded community trust [94].

The poster intervention also impacted self-efficacy. Women in Malaysia and participants (regardless of gender) in Lebanon who saw the posters, endorsed greater relationship problem-solving efficacy, including confidence in one’s ability to effectively seek solutions to IPV. 

Combined, results indicate that both interventions have the potential to encourage individual and community level problem solving around IPV. Results may be partially explained by the participatory approach taken for both interventions, including having community members facilitate the workshops and having workshop participants create the posters. Practitioners should consider similar participatory methods to build individual and community confidence around problem solving for IPA.

### 4.6. Mental Health, Functioning, and Coping

In both settings for both women and men, the workshop was associated with significant improvement in mental health symptoms and related functional impairment, and increased use of adaptive coping strategies. Use of violence as a coping mechanism (including arguing with partner and beating children in both settings and use of physical violence with partner in Lebanon) also decreased. This result may be explained by both the direct mental health and coping content woven throughout the 3 day workshop and the focus on social support, social cohesion and individual and community efficacy, all factors that have been associated with improved mental health and wellbeing [95]. 

The poster campaigns did not directly focus on mental health as a hypothesized outcome (rather, mental health data were collected as a demographic variable). However, by discouraging acceptability of violence and encouraging help-seeking for IPV, posters do attempt to indirectly target mental health. Because these downstream mental health consequences, such as help-seeking, might only be expected over a longer timeframe, it is not surprising that there were no significant differences in reported mental health symptoms by condition. 

Results underscore the value of incorporating mental health components into IPV group-based interventions, as research indicates that IPV can impact mental health, and poor mental health can undermine help-seeking [42,43,44] and increase risk of perpetrating IPV [48].

## 5. Limitations, Future Research, and Conclusions 

In Study 1, the workshop intervention shows significant promise based on the results presented here. However, interpretability of the workshop results is limited by the lack of a comparison group/counterfactual. Future research is needed with a more rigorous research design to assess effectiveness of this compelling intervention model.

Workshop outcomes were measured two weeks after the intervention. This is a strength in that many similar workshop models assess impact on the day of the workshop. However, it is not clear how long beyond the two weeks intervention impacts may last. Additional longitudinal work is needed to explore this further.

Other methodological considerations include duration of exposure in the poster condition in Study 2. Participants viewed the poster for a short period of time in a specific setting (at their place of residence, with a community interviewer). A few days later, a separate interviewer, blind to condition, visited to administer the follow up survey. While finding effects with such a short exposure is a strength, additional research should investigate the impact of a longer more naturalistic poster exposure, for example posters on public transport, or in other high traffic public places. It would also be useful to investigate poster impacts beyond a few days to see if such campaigns can result in lasting change.

The workshop and poster study designs included a limited number of intervention variants, and it is therefore difficult to determine the active ingredients influencing outcomes. For example, future research might compare the mental health-integrated IPV workshop to an IPV workshop that does not explicitly incorporate mental health. Similarly, perhaps the social norms-based approach or the participatory components are drivers of outcomes in one or both studies. Future work should attempt to isolate such mechanisms of change.

Finally, moving forward, it is important to unpack the specific benefits of the interventions by gender and for victims (usually women) and perpetrators (usually men). In Malaysia, for example, differences in results for women and men may be explained by the specific poster viewed. Men viewed a poster in Malaysia depicting a family experiencing IPV, whereas woman viewed a poster featuring a women considering various help-seeking options.

Context and cultural factors should also continue to be considered throughout the process of intervention development and evaluation. This includes matching methods to context. For example, to avoid potential contamination, a cluster comparison was considered feasible in Lebanon, whereas in Malaysia a randomized controlled trial (RCT) was not problematic. Utilizing a culturally specific lens will also assist in understanding surprising results in some settings (such as the ‘backlash effect’ in Lebanon), allowing for meaningful adaptation of interventions across a wide range of settings. This study explored the process of participatory co-creation and testing of two IPV interventions with Syrian refugees in Lebanon and Rohingya in Malaysia. Results of the workshop and community designed poster campaigns suggest that participatory, social norms-based, mental health-integrated intervention approaches can be utilized across cultures, with both women and men, and with victims and perpetrators. As such, this work has implications for service providers and researchers interested in community- generated solutions to IPV and can provide a roadmap for use in other settings.

## Figures and Tables

**Table 1:** Study 1 workshop interview measures.

Indicator	Measure	Description
**Demographics**	Investigator developed	Pre-interview: gender, age, partner status, age when married, number of children, people in the household, education level, employment.
**Prevalence of IPV**	10 items adapted from the short form of the Revised Conflict Tactics Scale-2 [78]	Measure of both perpetration and victimization of IPV, including psychological aggression, physical assault, injury and destruction of property. Results represent report of at least one incident of IPV in the past year.
**Acceptability of IPV**	WHO Multi-Country Study on Women’s Health and Domestic Violence, Women’s Questionnaire, Section 6 [3]	10-item scale, *Does a man have a good reason to hit his wife if…?* Followed by a brief scenario (e.g., *she does not complete housework to satisfaction*). Respondents indicate “disagree” or “agree”. Cronbach’s alpha: Malaysia, pre: 0.86, post: 0.92; Lebanon, pre: 0.85, post: 0.67.
**Beliefs about gender relations**	20 items adapted from Community Ideas about Gender Relations section of Attitude and Relationship Control Scales for Women’s Experiences of Intimate Partner Violence [79,80]	Measure of (1) respondents’ perceptions of community beliefs and (2) respondents’ personal beliefs. Includes four investigator-added items: *My community thinks/I think…that if a woman is abused by her partner, this is her fate; that people experiencing abuse by their partners should keep it to themselves*. Separate Community and Individual belief scales are presented (5-point response scale, 1 = strongly disagree; 5 = strongly agree).
**Attitudes toward help-seeking**	Three investigator-developed items assessing willingness to seek help for mental health and IPV victimization/perpetration	*If you were… feeling very sad and overwhelmed by difficulties; being abused by your partner/found that you were using abusive behaviors with your partner …would you tell someone/seek help to try to change this behavior?* (5-point scale, 1 = definitely no; 5 = definitely yes).
**Mental health**	13 items from symptom checklist of Refugee Health Screener (RHS)-15 [81]	Measure of symptoms of anxiety, depression, and post-traumatic stress disorder over the past two weeks (5-point scale, 0 = not at all; 4 = extremely). Cronbach’s alpha: Malaysia, pre: 0.91, post: 0.90; Lebanon, pre: 0.86, post: 0.91
**Functional impairment**	Three investigator-developed items	Report of difficulty (1) *Performing the tasks you need to do for your daily work*; (2) *Taking care of your family members*; (3) *Interacting with others socially* over the past two weeks (3-point scale, 1 = not at all difficult; 3 = very difficult).
**Social cohesion**	Five items adapted from Samson, Raudenbush, and Earls [82]	E.g., *People in this (Syrian/Rohingya) community are willing to help their neighbors* (5-point scale, 1 = strongly disagree; 5 = strongly agree).
**Self-efficacy**	Five items adapted from the Generalized Self-Efficacy Scale [83]	E.g., *I can always manage to solve difficult problems if I try hard enough* (4-point scale, 1 = not at all true; 4 = exactly true).
**Community-efficacy**	Two investigator-developed items	(1) *When confronted with relationship conflicts leading to violence, my community can find solutions*; (2) *Thanks to the resourcefulness of my community, we know how to handle unforeseen events such as violence arising from relationship conflicts* (4-point scale, 1 = not at all true; 4 = exactly true).
**Coping**	Six items adapted from the Brief COPE [84], and four investigator-developed items	Brief COPE items presented as a composite variable. Investigator items assessing coping through violence and self-soothing presented separately (4-point scale).
**Perceived workshop impact**	Five investigator-developed items	Assessment of impact of workshop participation over the past few weeks (see items in Results section) (4-point scale, 1 = strongly disagree; 4 = strongly agree).
**Workshop satisfaction**	7-item satisfaction survey in Lebanon and a 6-item survey in Malaysia, both collected immediately after the workshop.	Satisfaction with *clarity of information, communication of facilitators, training materials, questions answered, time of workshop, likelihood of advising others to participate in similar sessions or convey knowledge and skills to others* (5-point scale, 1 = strongly disagree; 5 = strongly agree).
**Reactions to research participation**	Items from the Reactions to Research Participation Questionnaire (RRPQ) [85]	12 items collected; for brevity’s sake, three items are presented (see items in Results section) (5-point scale, 1 = strongly disagree; 5 = strongly agree).

**Table 2:** Descriptive statistics, workshop participants.

	Malaysia			Lebanon		
	Total	Women	Men	Total	Women	Men
Sample, *n*	72	35	37	74	41	33
Age, Mean (SD) Range	31 (10.76) 18–59	30 (10.55) 18–56	32 (10.99) 18–59	34 (9.62) 18–61	33 (10.44) 18–61	35 (8.55) 21–51
Partner status, married and living with partner, % (*n*/total *n*)	65.3 (47/72)	88.6 (31/35)	43.2 (16/37)	82.4 (61/74)	78.0 (32/41)	87.9 (29/33)
Age when married, Mean (SD) Range	20 (6.12) 12–42	17(4.17) 12–29	24 (6.58) 15–42	22 (5.21) 14–35	19 (4.32) 14–32	25 (4.61) 14–35
Number of children, Mean (SD) Range	2.97 (2.58) 0–11	2.31 (1.89) 0–8	3.92 (3.15) 0–11	2.9 (2.64) 0–12	3.06 (2.63) 0–12	2.77 (2.67) 0–12
Number of people in household, Mean (SD) Range	6.07 (2.80) 2–14	5.83 (2.24) 2–10	2.92 (3.26) 1–5	5.47 (2.34) 1–12	5.71 (2.32) 2–12	5.18 (2.37) 1–10
Employed, % (*n*/total *n*)	59.7 (43/72)	22.9 (8/35)	94.6 (35/37)	62.2 (46/74)	58.5 (24/41)	66.7 (22/33)
Education, primary or more % (*n*/total *n*)	59.7 (43/72)	45.7 (16/35)	73.0 (27/37)	87.8 (65/74)	82.9 (34/41)	93.9 (31/33)
Intimate Partner Violence Exposure (1 or More Instances in the Past Year)						
*Insulted, swore, shouted, or yelled*						
I did to my partner, % (*n*/total *n*)	40.8 (29/71)	31.4 (11/35)	50.0 18/36)	71.4 (50/70)	65.8 (25/38)	78.1 (25/32)
My partner did to me, % (*n*/total *n*)	43.7 (31/71)	60.0 (21/35)	27.8 (10/36)	57.1 (40/70)	60.5 (22/38)	53.1 (17/32)
*Sprain, bruise, cut, or felt pain the next day*						
I had because of fight with my partner, % (*n*/total *n*)	22.5 (16/71)	37.1 (13/35)	8.3 (3/36)	8.6 (6/70)	15.8 (6/38)	0.0 (0/32)
My partner had because of a fight with me, % (*n*/total *n*)	4.2 (3/71)	0.0 (0/35)	8.3 (3/36)	1.4 (1/70)	2.6 (1/38)	0.0 (0/32)
*Pushed, shoved, or slapped*						
I did to my partner, % (*n*/total *n*)	16.9 (12/71)	11.4 (4/35)	22.2 (8/36)	14.3 (10/70)	21.1 (8/38)	6.3 (2/32)
My partner did to me, % (*n*/total *n*)	25.4 (18/71)	45.7 (16/35)	5.6 (2/36)	10.0 (7/70)	15.8 (6/38)	3.1 (1/32)
*Punched, kicked, or beat up*						
I did to my partner, % (*n*/total *n*)	11.3 (8/71)	0.0 (0/35)	22.2 (8/36)	1.4 (1/69)	2.7 (1/37)	0.0 (0/32)
My partner did to me, % (*n*/total *n*)	18.3 (13/71)	37.1 (13/35)	0.0 (0/36)	2.9 (2/68)	5.3 (2/38)	0.0 (0/30)
*Destroyed something belonging to my partner or threatened to hit my partner*						
I did to my partner, % (*n*/total *n*)	7.0 (5/71)	5.7 (2/35)	8.3 (3/36)	31.9 (22/69)	35.1 (13/37)	28.1 (9/32)
My partner did to me, % (*n*/total *n*)	11.3 (8/71)	14.3 (5/35)	8.3 (3/36)	18.6 (13/70)	26.3 (10/38)	9.4 (3/32)

**Table 3:** Workshop outcomes (pre- to post-interviews).

Variable	Wilcoxon Result, Z	
	Malaysia	Lebanon
Acceptability of violence ^a^	6.58 ***	5.97 ***
Community ideas about gender relationships ^b^ (*My perception of what my community believes)*	3.42 ***	−2.00 *
Individual ideas about gender relations (*What I believe personally*)	7.28 ***	5.13 ***
Help-seeking for mental health needs ^c^	3.88 ***	5.25 ***
Help-seeking for victims of IPV	5.84 ***	3.95 ***
Help-seeking for perpetrators of IPV	5.28 ***	3.05 **
Mental health (distress symptoms)	−6.08 ***	−6.82 ***
Functional impairment	−6.22 ***	−5.45 ***
Self-efficacy	5.70 ***	4.63 ***
Community efficacy ^d^	5.54 ***	0.18
Social cohesion ^d^	2.98 **	1.46
Adaptive coping scale ^e^	6.16 ***	4.50 ***
Coping by arguing/yelling (to deal with tension/stress)	−4.06 ***	−2.67 **
Coping by hitting my partner (to deal with tension/stress) ^f^	−0.42	−2.17 *
Coping by hitting my children (to deal with tension/stress) ^g^	−3.57 ***	−4.46 ***
Coping by using calming exercises (to deal with tension/stress)	6.93 ***	5.97 ***

* = *p* < 0.05, ** = *p* < 0.01; *** = *p* < 0.001. See Methods section for measures used. ^a^ Higher scores = less acceptability of violence. ^b^ Higher scores = more equitable beliefs (for both gender relations scales). ^c^ Higher scores = more likely to seek help (for all help-seeking items). ^d^ In Lebanon, six participants who presumably did not identify as part of the Syrian community did not answer community efficacy or social cohesion items ^e^ Brief COPE items were also analyzed separately. In Malaysia, five individual coping items changed significantly from pre- to post-intervention (*p*’s < 0.001), while one, taking comfort in religion, showed trend-level improvement (*p* = 0.054). In Lebanon, there was a significant increase in coping by trying to improve one’s situation, decrease in coping by giving up, and decrease in coping by blaming oneself (*p*’s < 0.001). There were no significant changes in receiving emotional support, receiving help and advice from others, or taking comfort in religion. ^f^ In Lebanon, completed for a subset of participants currently partnered (*n* = 63). ^g^ In Lebanon, completed by a subset of participants with children (*n* = 53).

**Table 4:** Study 2 poster interview measures.

Indicator	Measure	Description
**Demographics**	Investigator developed	Same as Study 1 (see Table 1).
**Prevalence of IPV**	Adapted from the short form of Revised Conflict Tactics Scale-2 [78]	As in Study 1, 10 items used in Malaysia. In Lebanon, local team elected to use a subset of six items (see Table 5) to reduce interview length and resolve confusion over terminology in Arabic. Results indicate at least one incident of IPV in the past year.
**Mental health**	Symptom checklist from WASSS-6 [88]	5-item scale assessing frequency of fear, anger, lack of interest, hopelessness, avoidance during the previous two weeks (5-point scale, 1 = none of the time; 5 = all of the time). Cronbach’s alpha: Malaysia, 0.79; Lebanon, 0.80.
**Acceptability of IPV**	WHO Multi-Country Study on Women’s Health and Domestic Violence, Women’s Questionnaire, Section 6 [3]	Same as Study 1 (see Table 1). Cronbach’s alpha: Malaysia, 0.78; Lebanon, 0.76
**Beliefs about gender relations**	Adapted from Community Ideas about Gender Relations section of the Attitude and Relationship Control Scales for Women’s Experiences of Intimate Partner Violence [79,80]	16 items in Malaysia and 14-items in Lebanon. As in Study 1, four additional investigator-created items were included (see Table 1). Results presented from individual subscale only.
**Relationship efficacy**	Adapted from the Generalized Self-Efficacy Scale [83]	Three items in Lebanon and five items in Malaysia adapted to apply to relationships (e.g., *I can always manage to solve relationship problems if I try hard enough*) (4-point scale, 1 = not at all true; 4 = exactly true).
**Help-seeking intention, IPV victimization**	Investigator developed item	*If you were being abused by your partner, would you tell someone/seek help?* using a 5-point response scale (1 = definitely no; 5 = definitely yes).
**Help-seeking source preferences**	Investigator developed item	Those indicating “yes” to the above question asked to indicate where they would seek help from a 9-item list.
**IPV help-seeking and help-giving scenario**	Investigator developed item	A vignette was read to participant: *Now I will tell you a story… you hear Yusuf yelling at Rahima...* Participants were asked to indicate what they would do and responses coded in a dropdown list. In Malaysia, participants also asked: *Do you think Rahima will want to seek help?* (Yes, Maybe, No).
**Reactions to research participation**	Items from the Reactions to Research Participation Questionnaire (RRPQ) [85]	Three items as in Study 1 (see Table 1).

**Table 5:** Descriptive statistics, poster testing participants.

Variable	Malaysia, *n* = 240		Lebanon, *n* = 260	
	Women, *n* = 121	Men, *n* = 119	Women, *n* = 131	Men, *n* = 129
Gender, % (*n*/grand total *n*)	50.4 (121/240)	49.6 (119/240)	50.4 (131/260)	49.6 (129/260)
Age, Mean (SD) Range	31 (9.19) 18–58	32 (9.28) 19–61	32 (10.05) 18–65	37 (12.88) 18–65)
Partner status, married and living with partner, % (*n*/total *n*)	88.4 (107/121)	46.2 (55/119)	82.4 (108/131)	86.8 (112/129)
Age when married, Mean (SD), Range	18 (5.15) 12–55	24 (5.73) 13–46	18 (4.20) 13–37	23 (4.64) 13–35
How partner chosen, arranged ^a,b^, % (*n*/total *n*)	69.0(81/121)	52.0 (43/88)	55.4 (72/130)	62.0 (70/113)
Number of children at home	2.42 (1.63) 0–6	1.33 (1.43) 0–5	3.93 (2.46) 0–11	4.19 (2.69) 0–12
Employed, % (*n*/total *n)*	19.8 (24/121)	88.2 (105/119)	16.8 (22/131)	2.3 (3/129)
Education, primary or more % (*n*/total *n*)	55.4 (67/121)	75.6 (90/119)	19.1 (25/131)	30.2 (39/129)
Time living in host country 1–5 years, % (*n*/total *n*) 5–10 years, % (*n*/total *n*)	63.0 (75/120) 23.3 (28/120)	57.1 (68/119) 41.2 (39/119)	51.9 (68/131) 39.7 (52/131)	50.4 (65/129) 42.6 (55/129)
Mental health, Mean, (SD) Range	3.89 (1.01) 1–5	3.48 (0.90) 1–5	2.96 (1.27) 1–5	3.88 (0.76) 1.2–5
Intimate Partner Violence exposure (1 or more instances in the past year), ^b,c,d^ %				
*Insulted, swore, shouted, or yelled* I did to my partner, % (*n*/total *n*) My partner did to me, % (*n*/total *n*)	31.4 (38/121) 46.3 (56/121)	58.8 (70/119) 29.4 (35/119)	20.0 (26/130) 40.0 (52/130)	54.9 (62/113) 12.4 (14/113)
*Sprain, bruise, cut, or felt pain the next day* I had because of fight with my partner, % (*n*/total *n*) My partner had because of a fight with me, % (*n*/total *n*)	24.0 (29/121) 0.0 (0/121)	4.2 (5/119) 3.4 (4/119)	--	--
*Pushed, shoved, or slapped* I did to my partner, % (*n*/total *n*) My partner did to me, % (*n*/total *n*)	5.8 (7/120) 28.9 (35/121)	23.5 (28/119) 2.5 (3/119)	6.9 (9/130) 26.9 (35/130)	25.7 (29/113) 0.9 (1/113)
*Punched, kicked, or beat up* I did to my partner, % (*n*/total *n*) My partner did to me, % (*n*/total *n*)	0.8 (1/120) 14.2 (17/120)	9.2 (11/119) 1.7 (2/119)	--	--
*Destroyed something belonging to my partner or threatened to hit my partner* I did to my partner, % (*n*/total *n*) My partner did to me, % (*n*/total *n*)	5.8 (7/121) 14.1 (17/121)	12.6 (15/119) 6.7 (8/119)	4.6 (6/130) 16.9 (22/130)	6.2 (7/113) 2.6 (3/113)

^a^ In Lebanon, 22 women, representing 30.5% of those with arranged marriages, reported that they did not agree to the marriage (also reported by one man). In Malaysia, two women and one man reported the same. ^b^ In Lebanon, one woman and 16 men reported that they had never been partnered, so were not asked about how partner was chosen or about IPV. In Malaysia, all women were partnered, although 31 men had never been partnered so they were not asked how partner was chosen. ^c^ There were more men in Malaysia without current partners than women (or men or women in Lebanon) so IPV and all outcomes were also examined for the subgroup of men currently with partners (see Results narrative). ^d^ The team elected to include a subset of IPV items in Lebanon for brevity’s sake and to reduce participant burden.

**Table 6:** Poster testing results.

Variables	Malaysia, *n* = 240		Lebanon, *n* = 258	
	Women	Men	Women	Men
Sample, *n* Poster condition, *n*; control condition, *n*	121 59, 61	119 56, 63	130 63, 67	128 61, 67
**Acceptability of violence (scale)** Odds Ratio, 95% CI	0.60 ***, 0.47–0.76	*ns*	Condition x gender interaction 0.57 ***, 0.41 to 0.79	
**Individual beliefs about gender relations (scale)** Coefficient, 95% CI	*ns*	−0.12 ^ (*p* = 0.057), 0.25–0.00	*ns*	
**Relationship problem-solving efficacy (scale)** Coefficient, 95% CI	0.3 3 **, 0.08–0.58	*ns*	Condition controlling for gender 0.21 *, 0.01–0.41	
**Help-seeking personal** (*if you were being abused, would you seek help?*) Odds Ratio, 95% CI	1.77 ^ (*p* = 0.097), 0.89–3.51	*ns*	*ns*	
**Help-seeking personal, sources** (*if you were being abused, who would you seek help from?*) Odds Ratio, 95% CI	Religious Leaders: 2.14 *, 1.02–4.50 Social Institutions: 2.48 *, 1.20–5.13	*ns*	Condition x gender interactions Family: 2.83 *, 1.04–7.69 Partner’s family: 0.17 ***, 0.06–0.49	
**Help-seeking beliefs** (*people experiencing abuse should keep it to themselves*) ^a^ Odds Ratio, 95% CI	*ns*	*ns*	Condition controlling for gender 1.63 *, 1.04–2.56	
**Scenario: Help-seeking** *(Do you think woman will want to seek help?)* Odds Ratio, 95% CI	1.99 ^ (*p* = 0.051), 0.98–4.04	*ns*	*Item not used in Lebanon*	
**Scenario: Help-giving** (*React by not getting involved*) Odds Ratio, 95% CI	0.30 *, 0.11–0.83	*ns*	Condition controlling for gender 0.64 ^ (*p* = 0.097), 0.38–1.09	
**Scenario: Help-giving** (*React by talking to couple*) Odds Ratio, 95% CI	*ns*	*ns*	Condition x gender interaction Talk to wife: 0.35 *, 0.13–1.00 Condition controlling for gender Talk to husband: 1.80 *, 1.11–2.94	

*ns* = non-significant, ^ = *p* < 0.1, * = *p* < 0.01, ** = *p* < 0.01; *** = *p* < 0.001. Please see Methods section for tools used and Results narrative for interpretations of interaction effects and post hoc tests. Statistics for non-significant results are available upon request. ^a^ This was an investigator developed item (reverse coded) added to the gender relations scale specifically focused on help-seeking so it was examined separately here.

## Data Availability

Data are available upon request to Courtney.Welton-Mitchell@cuanschutz.edu.
